# Attomolar Detection of HIV‐1 With Label‐Free RCA‐rCRISPR on Smartphone

**DOI:** 10.1002/advs.202519508

**Published:** 2026-09-02

**Authors:** Noor Mohammad, Anastasiia Steksova, Yuyang Tang, Leyao Huang, Alireza Velayati, Shengwei Zhang, Aditi Dey Poonam, Sina Jamalzadegan, Matthew Breen, Hongjie Chen, Mahsa Bagi, Guochun Jiang, Qingshan Wei

**Affiliations:** ^1^ Department of Chemical and Biomolecular Engineering North Carolina State University Raleigh North Carolina USA; ^2^ Department of Chemical Engineering Bangladesh University of Engineering and Technology Dhaka Bangladesh; ^3^ UNC HIV Cure Center and Department of Biochemistry & Biophysics University of North Carolina Chapel Hill North Carolina USA; ^4^ Department of Molecular Biomedical Sciences College of Veterinary Medicine North Carolina State University Raleigh North Carolina USA; ^5^ Comparative Medicine Institute North Carolina State University Raleigh North Carolina USA

**Keywords:** CRISPR, HIV, minigel electrophoresis, plasmid DNA reporter, point‐of‐care diagnostics

## Abstract

HIV remains a major global public health challenge, causing 42.3 million deaths since its discovery in the early 1980s. Despite progress in prevention and treatment, around 60% of people with HIV (PWH) remain undiagnosed in resource‐limited regions due to the lack of inexpensive and equipment‐free detection methods. Here, we developed a low‐cost, robust, and label‐free CRISPR‐based diagnostic platform for detecting HIV viral load with minimal instrumentation. Our strategy combines rolling circle amplification (RCA) with plasmid reporter‐based ratiometric CRISPR (rCRISPR) that enables the detection of HIV RNA down to single‐digit aM sensitivity from PWH‐derived HIV samples ex vivo. Unlike conventional RCA, which requires fragmentations of long RNA target sequences, our design harnesses the triple functions of the phi29 DNA polymerase (namely exonuclease activity, polymerization, and strand displacement), enabling the detection of the long HIV genome without pre‐fragmentation. Cas12a reaction then detected RCA products by converting supercoiled ΦX174 plasmid reporters to relaxed forms. The target concentration was quantified based on the supercoil‐to‐relaxed plasmid ratio. Further, we constructed an all‐in‐one smartphone‐based minigel electrophoresis device to demonstrate equipment‐free HIV viral load testing. Finally, the assay has demonstrated for *BRAF* point mutation detection, showcasing the robustness of our strategy for broad disease diagnostic applications.

## Graphical abstract

A new diagnostic strategy integrating rolling circle amplification (RCA) with a plasmid reporter–based ratiometric CRISPR (rCRISPR) system was developed to detect unfragmented HIV genomes with single‐digit attomolar sensitivity. The method quantifies targets using the plasmid relaxation ratio and employs a smartphone‐based mini‐gel device to enable equipment‐free HIV viral load testing.

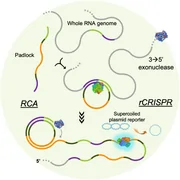

## 1 Introduction

Human Immunodeficiency Virus‐1 (HIV‐1) remains one of the most significant public health challenges globally, with approximately 40 million people living with the virus worldwide as of 2023 [1]. Since its discovery in the early 1980s, HIV has led to over 42.3 million deaths, making it a leading cause of morbidity and mortality worldwide [1]. Despite substantial progress in prevention and treatment efforts, around 60% of people with HIV (PWH) are going undiagnosed in resource‐limited regions [2], which means the resulting disease disproportionately affects vulnerable populations and underserved communities, highlighting the urgent need for accessible and accurate point‐of‐care (POC) diagnostic tools to combat the spread of the virus.

The traditional HIV diagnosis relies on the use of antigen/antibody or polymerase chain reaction (PCR) testing [3]. Antibody/antigen tests suffer from low specificity and lack the sensitivity required for reliable early detection, whereas PCR is costly, time‐consuming, complex, laborious, and requires a specialized laboratory and skilled technical staff. The technological advancement of gene‐editing using CRISPR (Clustered Regularly Interspaced Short Palindromic Repeats) with associated proteins (aka. CRISPR‐Cas) [4, 5, 6, 7] offers promising opportunities for the development of rapid, sensitive, and specific molecular diagnostics [8, 9, 10, 11, 12]. CRISPR‐based diagnostics (CRISPR‐Dx) came into the limelight after the discovery of Cas13a‐based Specific High‐Sensitivity Enzymatic Reporter UnLOCKing (SHERLOCK) platform for detecting RNA sequences [9], and the Cas12a‐based DNA endonuclease‐targeted CRISPR *trans* reporter (DETECTR) mechanism to detect DNA targets [8]. Numerous CRISPR‐Dx platforms have been developed to date, showcasing the immense potential of CRISPR systems in addressing the longstanding challenge of achieving highly sensitive and specific nucleic acid detection through a comparatively rapid process [13, 14, 15, 16, 17, 18, 19, 20, 21, 22, 23, 24, 25, 26, 27]. CRISPR‐Cas12a particularly draws extensive attention, given its ease of reconfiguration for detecting a wide range of human, animal, and plant diseases [8, 14, 17, 20, 28, 29, 30, 31], including gene mutations [32, 33]. For signal acquisition purposes, CRISPR‐Cas12a‐Dx typically relies on fluorescent, colorimetric, or electrochemical readout [8, 9, 13, 18, 24, 26, 29, 32, 34, 35, 36, 37, 38, 39, 40, 41]. The signal is mainly achieved when CRISPR‐Cas12a nonspecifically cleaves the reporter molecules (typically ssDNA) via its unique *trans*‐cleavage mode and changes the fluorescence [8, 9, 13, 18, 26, 32, 34, 36, 37, 40], color [29, 35, 38, 39], or current states [24, 41] of the reporters. We previously demonstrated a simple, inexpensive, and nonoptical CRISPR‐Cas12a‐based sensing platform to detect DNA targets at sub‐picomolar (pM) detection sensitivity using double‐stranded lambda (ds λ) DNA [42], hybrid DNA (i.e., dsDNA backbone plus a 3’ toe) [43], and ds plasmid DNA [44] as label‐free reporters. These assays quantify target concentrations by measuring reporter size changes. The plasmid reporter is especially appealing for POC applications due to its combined benefits of cost‐effectiveness, rapid reaction kinetics, high detection sensitivity, and ratiometric signal readout through molecular sizing to reduce size errors [44].

While Cas12a has been shown to recognize DNA targets, recent studies employing techniques such as split crRNA or Split Activator for Highly Accessible RNA Analysis (SAHARA) have demonstrated the detection of RNA targets using CRISPR‐Cas12a [31]. However, RNA detection using these methods often suffers from low detection sensitivity. More recently, researchers combined RCA with fluorescently labeled CRISPR‐Cas12a, which enabled the detection of miRNA at single‐digit femtomolar (fM) levels [30]. While this approach is effective for miRNA detection, detection of an entire unprocessed viral genome, such as HIV, at clinically relevant levels (low attomolar, or aM) remains challenging. Moreover, the development of a low‐cost device for assay readout is crucial if such an assay is to offer a significant impact for adoption in resource‐limited communities.

**SCHEME 1:**
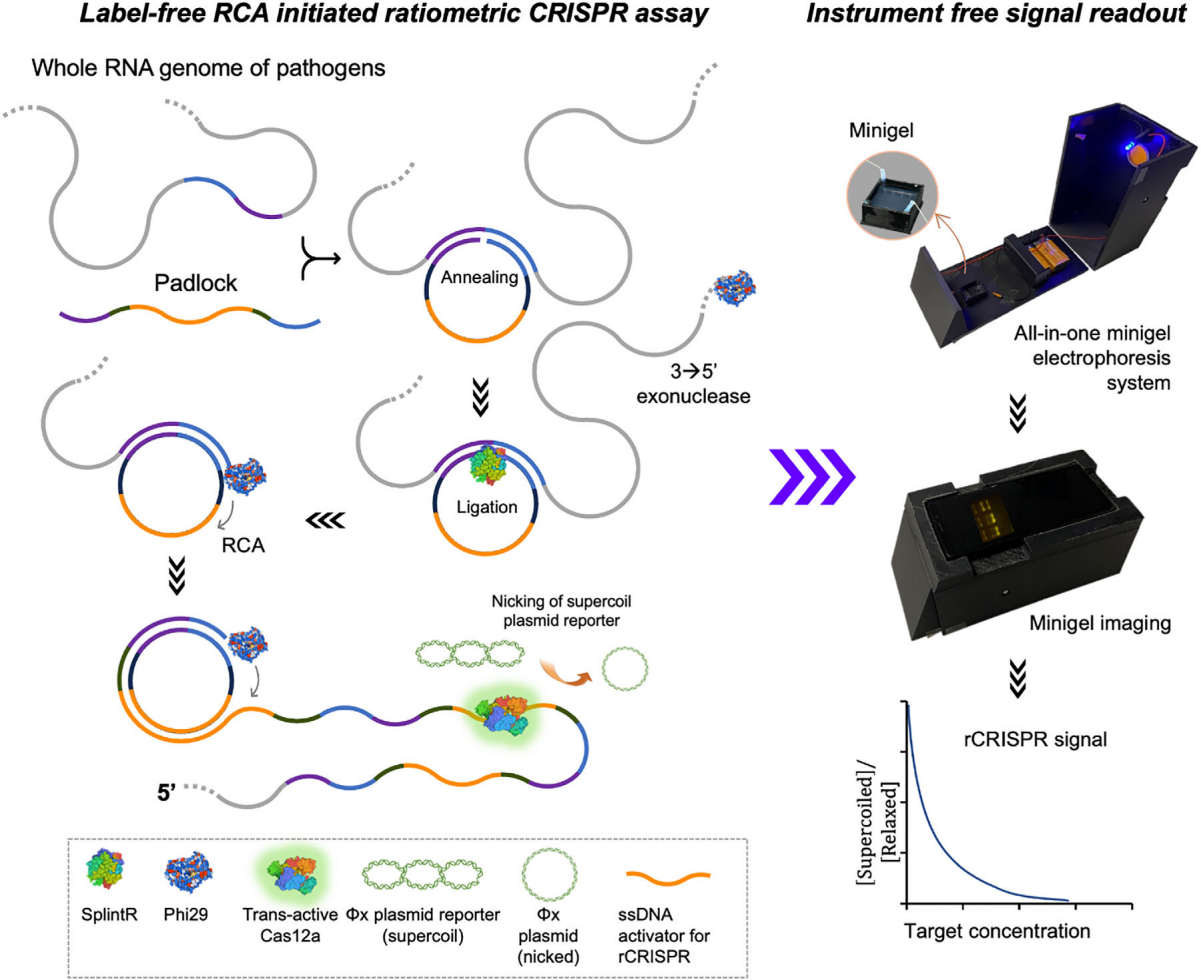
Schematic of plasmid reporter‐powered RCA‐rCRISPR integrated with a smartphone reader for detecting unprocessed, long HIV genome at POC. The padlock sequence does not contain the target sequence; rather, it includes the reverse complement (purple and blue ends) of the target (22 nt). The black components in padlock are arbitrary spacer sequences that do not participate in RCA; however, they help prevent the padlock sequence from forming secondary structures. Moreover, the orange‐colored sequence represents both the rCRISPR activator sequence (in RCA product) and its reverse complement (in padlock).

Here, we developed a low‐cost, robust, and label‐free RCA‐rCRISPR diagnostic platform coupled with a smartphone readout device for detecting the unfragmented HIV genome derived from PWH ex vivo (Scheme 1). This innovation combines a plasmid‐based ratiometric CRISPR (rCRISPR) strategy with RNA‐detecting RCA. Amplicon DNA generated by the RCA step activates Cas12a, which induces *trans*‐nicking of unlabeled supercoiled ΦX174 (5.37 kb) [45] plasmid DNA reporter, generating a sensitive ratiometric signal (the ratio of supercoiled to circular plasmid). The assay signal is then quantitatively measured by a portable molecular sizing device such as the smartphone‐based mini gel system. Our label‐free CRISPR diagnostic platform detected HIV viral load as low as approximately 3000 copies/mL (∼5 attomolar) in PWH‐derived HIV ex vivo, which is approximately two orders of magnitude greater sensitivity compared to typical RCA‐integrated fluorescent CRISPR methods (Table S2). In contrast to conventional RCA methods that necessitate sample pretreatment to detect lengthy genomic RNA, our design harnesses the triple functions of phi29 protein (fast exonuclease activity, polymerization, and strand displacement), enabling the detection of the entire HIV genome without pre‐fragmentation. For point‐of‐care (POC) applications, we have developed an integrated minigel electrophoresis device coupled with a smartphone‐based fluorescence reader, enabling equipment‐free testing of HIV viral load. Furthermore, our assay exhibits versatility by successfully detecting point mutations, such as the *BRAF* mutation associated with canine urothelial cancer [46, 47, 48, 49], highlighting the robustness and broad applicability of our diagnostic strategy across various diseases.

## 2 Results and Discussion

### 2.1 Integrating Label‐Free rCRISPR With RCA for RNA Detection

**FIGURE 1:**
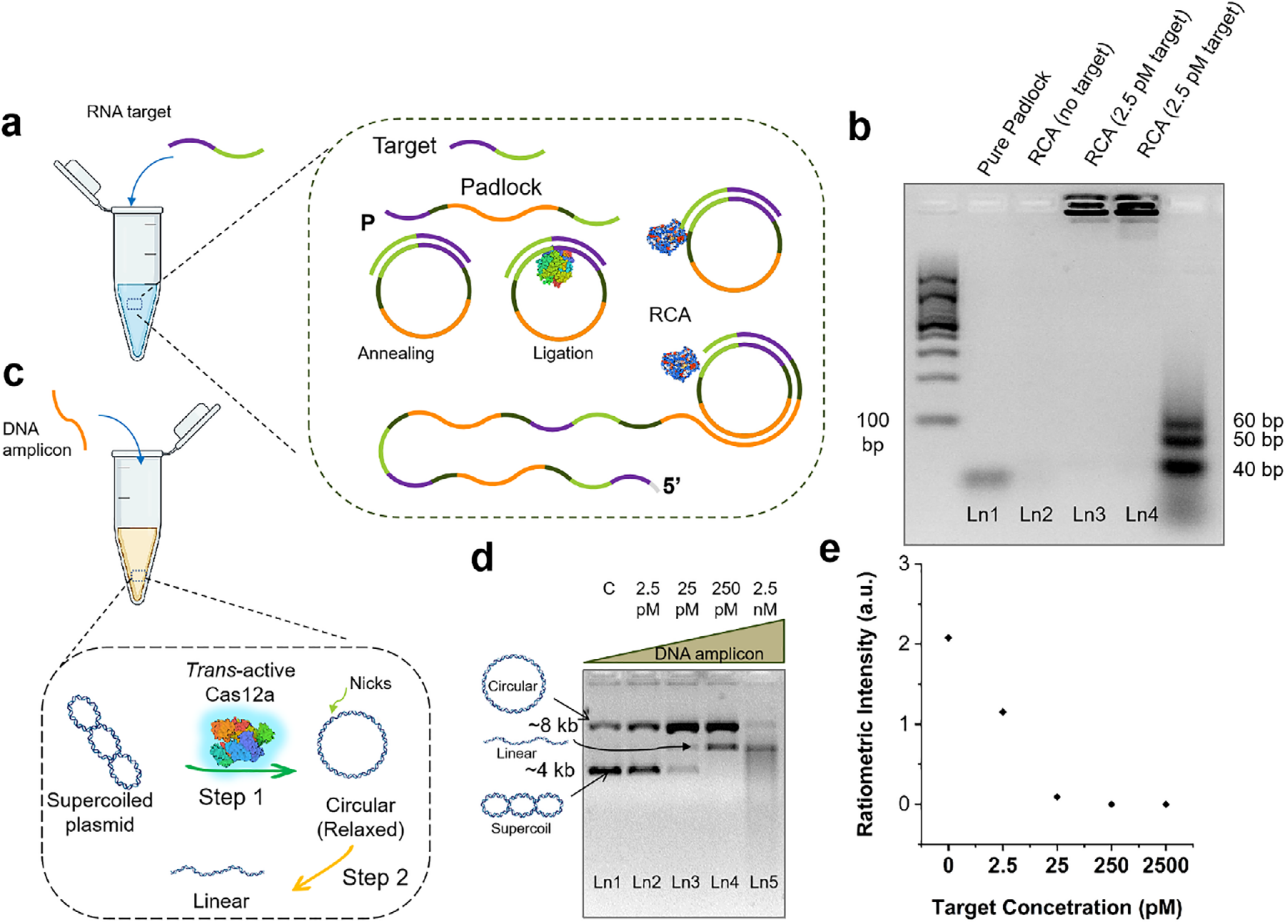
Demonstration of RCA and label‐free rCRISPR separately. (a) Schematic of RCA for RNA detection. The padlock sequence does not contain the target sequence; rather, it contains the reverse complement of the target. In the figure, the orange‐colored sequence represents both the target sequence and its reverse complement. (b) Gel electrophoresis (4% agarose gel and 1×TBE buffer) results demonstrating results for RCA assay performed at room temperature for 2 h. (c) Schematic demonstration of label‐free rCRISPR for ssDNA detection using ΦX174 plasmid reporter. (d) Gel electrophoresis (1% agarose gel and 1×TBE buffer) results demonstrating rCRISPR assay performed at 37°C for 1 h. Ln1: negative control; Ln2‐5: increasing concentrations of target. Additional gel data with band sizes are in Figure S1b. (e) Ratiometric signal achieved from rCRISPR strategy. Abbreviations: RCA, Rolling circle amplification; c, negative control; nM, nanomolar; pM, picomolar; ssDNA, single‐stranded DNA; bp, base pair; Ln, lane; TBE, Tris‐borate EDTA; a.u., arbitrary unit.

rCRISPR is a novel CRISPR‐Cas12a‐based diagnostic method for detecting DNA targets using a label‐free plasmid reporter molecule [44], and the RCA assay is an isothermal nucleic acid amplification method. rCRISPR is constructed based on the relaxation of plasmid DNA reporter, which is triggered by Cas12a‐mediated nicking in the presence of the target. When the *trans*‐active Cas12a cleaves the supercoiled plasmid, it relaxes into a circular form (Figure S1). With further nicking, the circular plasmid gradually converts into a linear form (Figure 1c). While the linearization process (step 2) occurs relatively slowly, our previous study demonstrated that the conformational change from supercoiled to circular DNA (step 1) is very rapid [44]. This relaxation process can be quantified ratiometrically by measuring the ratio of supercoiled to circular plasmid to determine the DNA target concentration. The combination of these two techniques, namely RCA and rCRISPR allows us to expand the detection capability of rCRISPR from DNA targets to RNA.

We first tested the RCA and rCRISPR assays separately and studied the feasibility of detecting RNA molecules using a label‐free plasmid reporter. Figure 1a shows the mechanism of RCA assay. We designed an RCA padlock to detect a 22 nt long synthetic RNA target (sequences of padlock and 22 nt RNA target are in Table S1). The 3’‐end of the target has reverse complementarity to the 5’ end of the padlock, and the 5’‐end of the target has reverse complementarity to the 3’ end of the padlock. This complementary matching design helps the target nucleic acid bind to both ends of the padlock and join the two ends of the padlock together. The SplintR ligase enzyme then ligates both ends of the padlock and produces a circular template, on which the target RNA acts as the primer for the RCA assay. Next, the phi29 DNA polymerase starts synthesizing DNA in a 5’ to 3’ direction. With a unique ability to displace the downstream DNA strand, phi29 continues DNA synthesis even when encountering secondary structures or other obstacles in the template DNA. In this manner, phi29 keeps rolling over the circular template and produces many copies of reverse complementary sequences of padlock with the long ssDNA product >10 knt. We performed RCA for 2 h at room temperature and then ran gel electrophoresis to evaluate the reaction products. Figure 1b (lanes 3 and 4) shows that in the presence of RNA targets, RCA reaction occurred successfully. We observed large molecular weight DNA products (bands) adjacent to the wells, confirming the presence of RCA products. In contrast, in the absence of RNA targets, no amplification was noticed (Figure 1b, lane 2). These results demonstrated that an RNA‐detecting RCA assay was successfully constructed, consistent with prior studies [30].

Next, we confirmed the feasibility of detecting ssDNA using rCRISPR with a ds plasmid reporter (supercoiled ΦX174). To demonstrate the rCRISPR assay with ΦX174 supercoiled plasmid reporter, we first activated the Cas12a with a 20 nt long ssDNA (see sequence in Table S1), and added the plasmid reporter in the reaction. Figure 1c shows the schematics of a label‐free CRISPR‐Cas12a assay using a supercoiled plasmid reporter. We performed the assay with 2.5 pM, 25 pM, 250 pM, and 2.5 nM of targets, with a negative control. After 1 h of reaction, the gel electrophoresis showed that supercoiled bands became thinner for all of the concentrations except for the negative control (Figure 1d). We measured band intensities (Figure S2), and plotted ratiometric signals (ratio of supercoil to circular band) against different target concentrations. Figure 1e shows that with the increase of target concentrations, the ratiometric signal went down and became zero for higher target concentrations above 25 pM.

**FIGURE 2:**
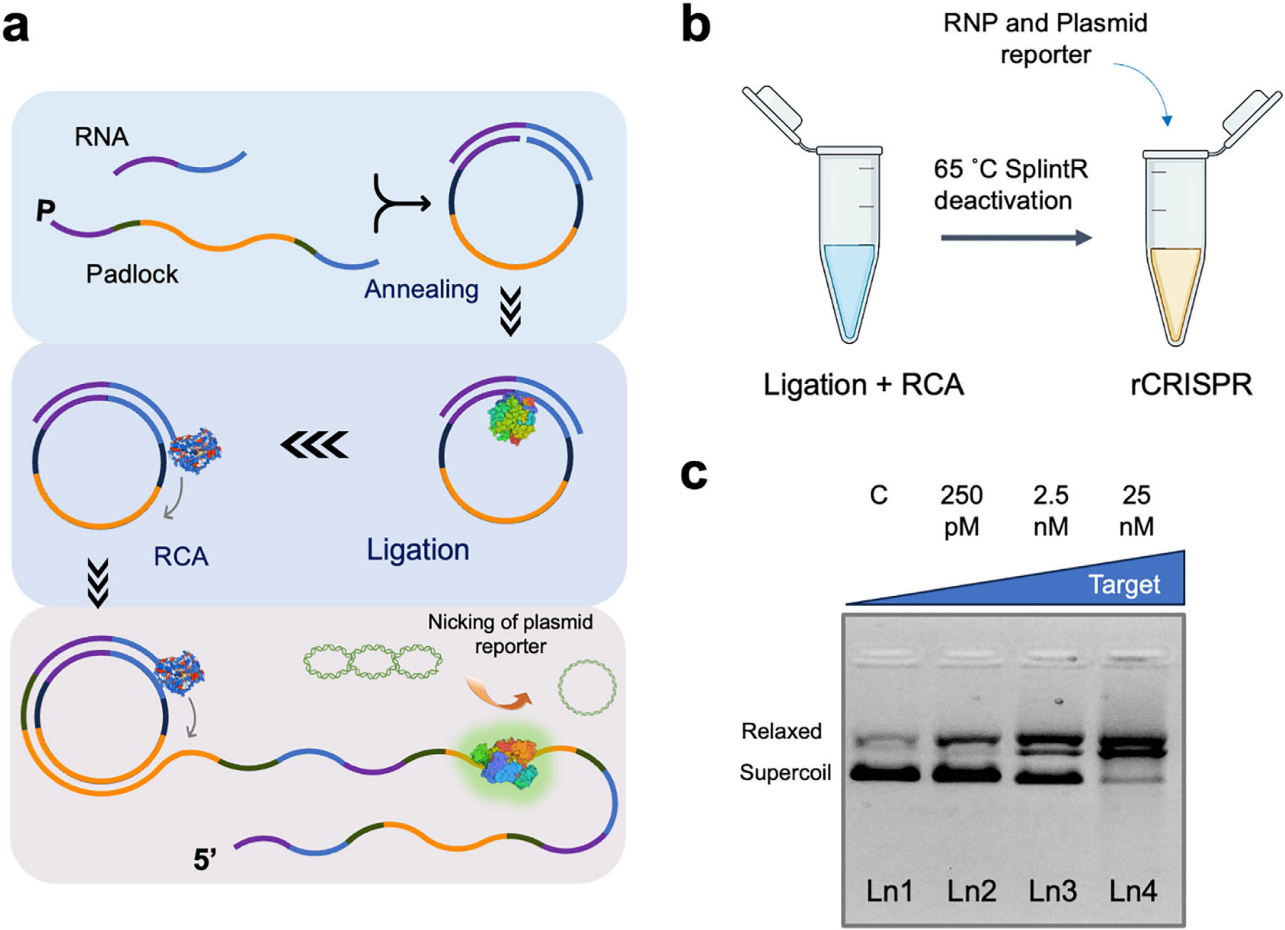
RNA detection using RCA‐rCRISPR. (a) Mechanism of RCA‐integrated rCRISPR. (b) Schematic of SplintR denaturation. (c) Gel electrophoresis (1% agarose gel and 1×TBE buffer) results demonstrating RNA detection using RCA‐rCRISPR assay. Total reaction time was 3 h, of which 2 h were used for RCA and 1 h for rCRISPR. Both reactions were performed at 37°C. RCA, Rolling circle amplification; c, negative control; nM, nanomolar; pM, picomolar; Ln, lane; TBE, Tris‐borate EDTA.

Then, we combined both assays together to detect RNA targets (Figure 2a). We mixed all the required reagents for RCA and rCRISPR in a single pot and incubated the reaction mixture for 2 h at 37°C. We performed the assay with RNA targets of 250 pM, 2.5 nM, 25 nM concentrations, and 0 target (negative control) to evaluate the feasibility of RCA‐rCRISPR scheme for RNA detection. Figure S3a shows that in the initial trial, we didn't observe any supercoil relaxation for any of the target concentrations. After careful troubleshooting of all assay conditions, we attributed the failure of the direct combination of RCA and rCRISPR reactions to the opposite functionality of SplintR ligase and Cas12a enzyme. We suspected that while target‐activated Cas12a tended to nick and unwind supercoiled plasmid reporters, the SplintR ligase on the other side would repair the Cas12a‐induced nicks and recover its supercoiled status (Figure S3b). One potential way to circumvent this problem is to deactivate the SplintR ligase before the subsequent rCRISPR reaction. To test that, we first ran the RCA reaction at room temperature and then deactivated the ligase protein at 65°C before performing the rCRISPR at 37°C. The procedure of the assay and gel results are shown in Figure 2b,c, respectively. The gel results showed a positive rCRISPR assay result this time, confirming that Cas12a‐induced DNA supercoil relaxation happened after thermal denaturation of RCA ligase.

**FIGURE 3:**
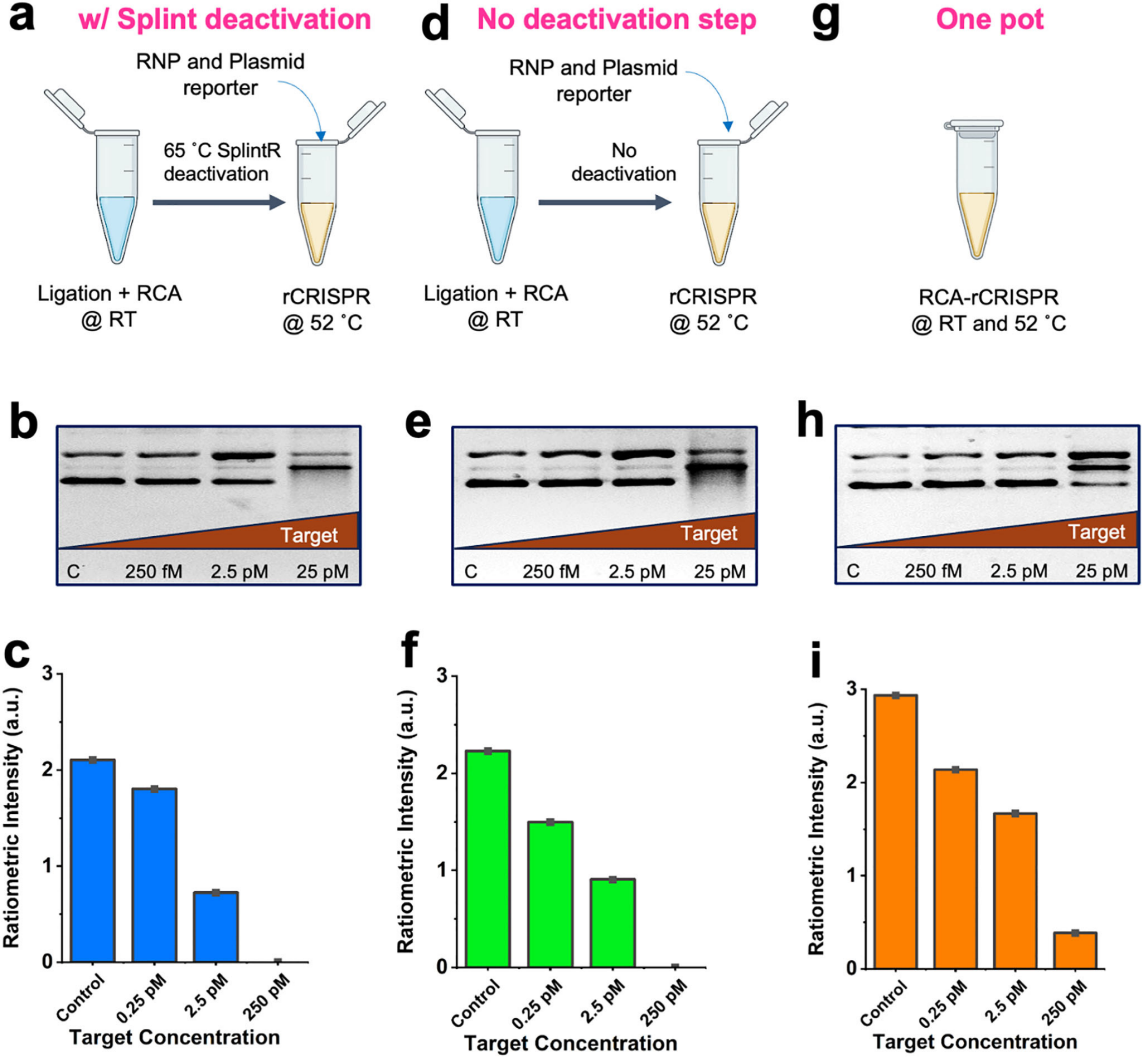
Assay simplification. Schematics show experimental procedures with ligase deactivation (a), without ligase deactivation (d), and one‐pot assay (g). Gel electrophoresis (1% agarose gel and 1×TBE buffer) results demonstrating RNA detection using rCRISPR with ligase deactivation (protocol i) (b), without ligase deactivation (protocol ii) (e), and one‐pot assay (protocol iii) (h). Histogram showing quantitative ratiometric signals for the assays with ligase deactivation (protocol i) (c), without ligase deactivation (protocol ii) (f), and one‐pot assay (protocol iii) (i). Corresponding intensity diagrams are shown in Figure S6. Total reaction time was 3 h, of which 2 h were used for RCA and 1 h for rCRISPR. Abbreviations: RCA, Rolling circle amplification; RT, room temperature; c, negative control; pM, picomolar; Ln, lane; TBE, Tris‐borate EDTA; a.u., arbitrary unit.

To simplify the assay protocols and explore the possibility of combining two separate assays in one pot, instead of deactivating SplintR, as ATP is an essential molecule for ligation assay, and can be degraded at temperatures in excess of 40°C [50, 51], we chose to inactivate ATP once ligation is complete. On the other side, rCRISPR can be performed at higher temperatures (>45°C). The idea is to perform rCRISPR at an ATP‐susceptible temperature (e.g., 52°C) to inhibit plasmid reporter repair during the CRISPR reaction. To test the idea, we designed three different assay protocols: (i) two‐step RCA‐rCRISPR with SplintR deactivation at 65°C, (ii) two‐step RCA‐rCRISPR at 52°C without SplintR deactivation step, and (iii) one pot RCA‐rCRISPR assay at 52°C (Figure 3a,d,g).

For protocol i, the assay worked as expected (Figure 3a–c). For protocol ii (without SplintR deactivation), the RCA assay tubes (without CRISPR reagent) were kept at RT for 2 h and then CRISPR reagents were added. The tubes were incubated at 52°C for 1 h. For protocol iii (one‐pot assay), we combined everything in one tube, kept it at RT for 2 h and then moved it to an incubator at 52°C. The concept is that the ligation reaction would be favorable at a lower temperature [52]. At a higher temperature, ATP will be degraded, thus inhibiting the ligation reaction [51] and leading to a positive rCRISPR signal. Qualitative gel results (Figure 3b,e,h) and quantitative ratiometric signal measurement (Figure 3c,f,i) showed the success of all three assay protocols. However, if we compare the supercoil bands for the 250 pM targets in Figure 3b,e, we notice that protocol ii (without deactivation) generated the strongest signal. Therefore, we followed protocol ii for all future experiments. We also evaluated the effect of padlock concentrations on the final assay signal intensity (Figure S4) and found that the higher padlock concentration (250 nM) hampered the overall assay signals. This might be due to the binding of two padlocks to one target when the padlock concentration is too high. This side effect would reduce the formation of the desirable circular templates for RCA and therefore reduce signal intensity (Figure S5).

### 2.2 HIV Detection Using the RCA‐rCRISPR Technique

**FIGURE 4:**
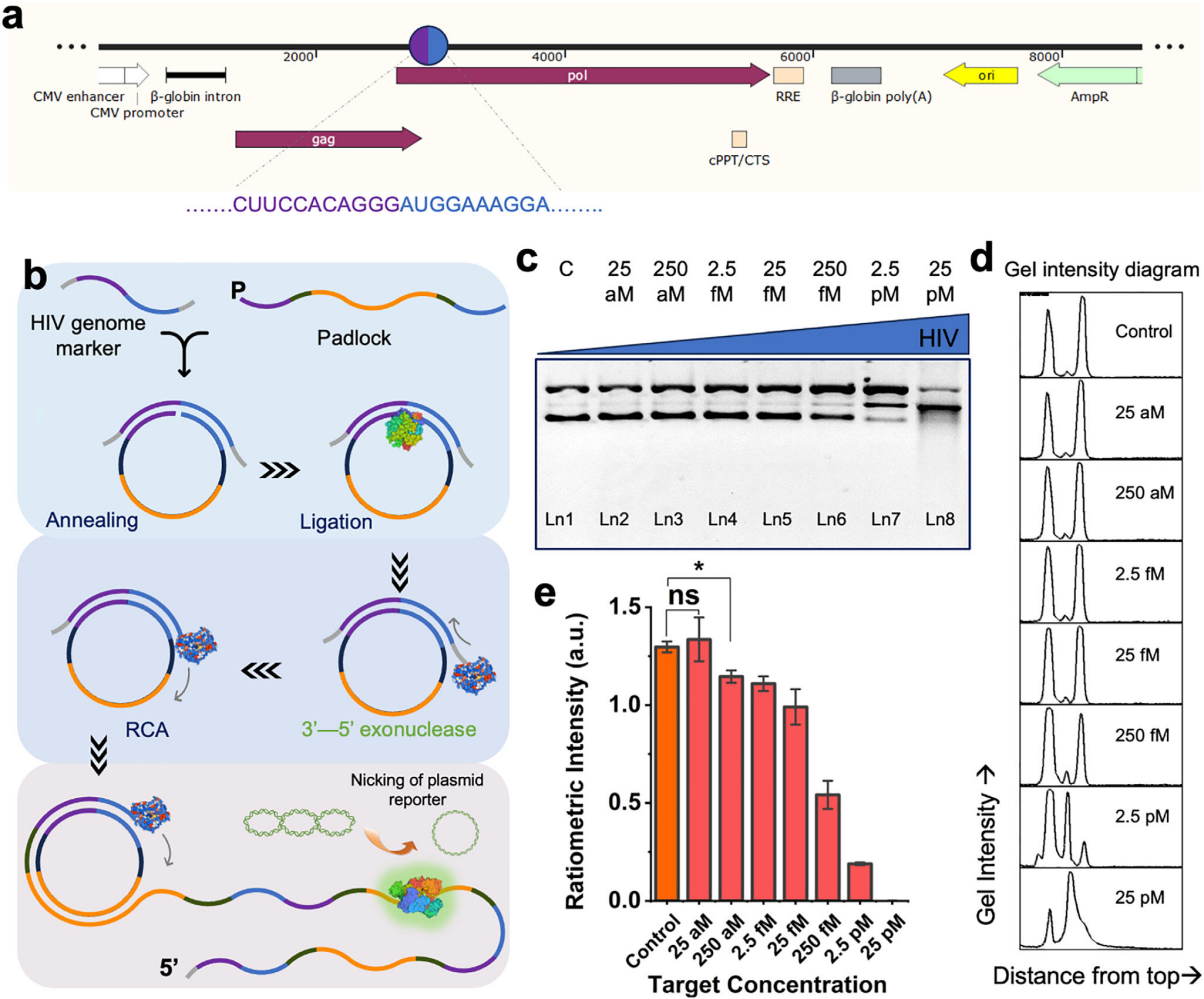
Synthetic HIV RNA (pol gene) detection using RCA‐rCRISPR technique. (a) HIV RNA target selection. (b) Assay mechanism of HIV RNA detection utilizing RCA‐initiated rCRISPR technique. (c) Gel electrophoresis (1% agarose gel and 1×TBE buffer) demonstrating rCRISPR assay result for HIV RNA target detection. (d) Gel intensity diagrams of each lane in c. (e) Quantitative performance (LOD quantification for synthetic HIV RNA) of rCRISPR. Total reaction time was 3 h, of which 2 h were used for RCA and 1 h for rCRISPR. RCA was done at room temperature while rCRISPR was performed at 52°C. The graph shows statistical insignificance at *p* > 0.05 (ns), and significance at *p* < 0.05 (*). Abbreviations: c, negative control; aM, attomolar; fM, femtomolar; pM, picomolar; Ln, lane; TBE, Tris‐borate EDTA; a.u., arbitrary unit.

After optimizing and simplifying the assay, we tested the new RCA‐rCRISPR approach for HIV target detection. We chose the pol region of the HIV genome as our target (Figure 4a, also see sequence in Table S1) [53]. An RCA padlock was designed to recognize a 22 nt sequence from the pol region (see the sequence in Figure 4a). For the proof‐of‐concept demonstration, we synthesized a 28‐nt (3 nt extra on either side) sequence to mimic the target of the HIV genome. The extra nucleotides were added to evaluate the assay performance when the target was not the exact size of the hybridization site of the padlock.

Figure 4b shows the mechanism of detecting the 28 nt synthetic HIV target using the RCA‐rCRISPR strategy. The main difference in this schematic compared to the previous one in Figure 2a is the exonuclease activity of phi29. Here, since we have three extra nucleotides at the 3’ end of the target, we propose that when polymerization starts, these extra nucleotides will be removed by phi29. After that, the HIV target would act as the primer for RCA amplification. We performed the assay for a wide range of target RNA concentrations, ranging from 25 aM to 25 pM, including a negative control (Figure 4c,d). To evaluate the assay performance, we measured the limit of detection (LOD), which was found to be ∼250 aM, which is better than the detection result observed for the RNA target used in Figures 2 and 3. The sensitivity of RCA combined with CRISPR can vary depending on the target, as the probe binding efficiency may differ for different sequences [54]. This explains why we observe different results for the RNA targets used in Figures 2 and 3 compared to the HIV RNA target used in Figure 4.

**FIGURE 5:**
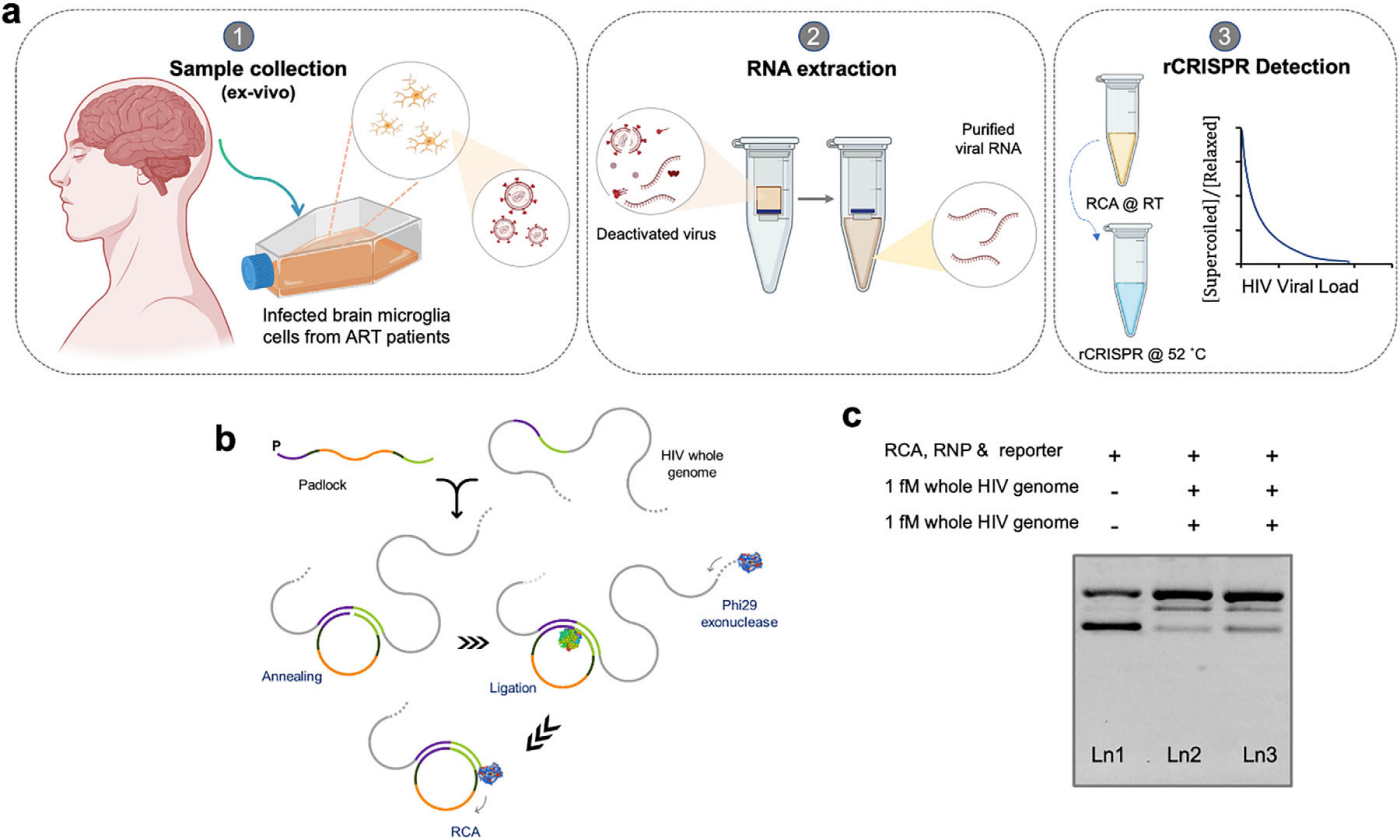
Ex vivo HIV RNA detection with RCA‐rCRISPR. (a) Schematic showing the overall procedures of detection of the HIV genome from PWH. (b) Mechanism showing how a very large viral RNA (i.e., long HIV genome without ultrasonication) can initiate RCA reaction without a primer. (c) Gel electrophoresis (1% agarose gel and 1×TBE buffer) demonstrated rCRISPR assay for unfragmented HIV genome detection. Total reaction time was 3 h, of which 2 h were used for RCA and 1 h for rCRISPR. RCA was done at room temperature while rCRISPR was performed at 52°C. Abbreviations: fM, femtomolar; Ln, lane; TBE, Tris‐borate EDTA.

We next applied the optimized assay to measure HIV RNA in samples directly isolated from PWH. We obtained HIV samples, which were originally isolated from the brain tissue of PWH, from the HIV Cure Center at the University of North Carolina at Chapel Hill (UNC‐Chapel Hill). The donor was undergoing antiretroviral therapy (ART) and had an undetectable plasma viral load [55]. Therefore, instead of attempting direct detection, we used PWH‐derived HIV samples to perform *ex vivo* infection of primary brain microglia. Our data showed that we were able to detect the viral RNA release in culture supernatants in this ex vivo infection study. Prior to performing RCA‐rCRISPR, we conducted RT‐digital droplet PCR (ddPCR) experiments to quantify the amount of HIV genome and p24 antigen in the samples. The viral genome load was found to be in the femtomolar range (converting “copies/mL” to “molarity” yields, 1.3 fM‐1.7 fM) (Figure S7a). Using these quantified data, we calculated the viral genome concentration in the samples, which was then used in the subsequent RCA‐rCRISPR experiments. Figure 5a demonstrates the steps involved in the detection of PWH‐derived HIV RNA using label‐free RCA‐rCRISPR. Briefly, the collected viruses from the cultured supernatants were lysed in 1% Triton X‐100 to release their viral load. Viral RNA was purified using a spin column and then added to the label‐free RCA‐rCRISPR assay. Figure 5b shows the 3′→5′ exonuclease activity of phi29 to trim the long unfragmented HIV genome (∼9 kbp long) and subsequent RCA amplification. The size and sequence of the extracted HIV sample were confirmed in one of our previous study [56], and can also be found in GenBank (accession # OQ325479, or https://www.ncbi.nlm.nih.gov/nuccore/OQ325479). Figure 5c shows the assay result with the unfragmented HIV genome from the PWH‐derived HIV samples. Lane 1 was for control, and lanes 2 and 3 were for the assay with samples containing PWH‐derived unprocessed HIV. To confirm that these results are not false positives, we first verified that the viral target sequence we chose did not match the human genome (Figure S8a). In addition, since we know that our label‐free assay is based on the *trans‐*nicking of the plasmid reporter, we ran assays with and without the padlock to confirm that the supercoil relaxation in Figure 5c is not due to unknown interferences from samples with PWH‐derived HIV (Figure S8b).

**FIGURE 6:**
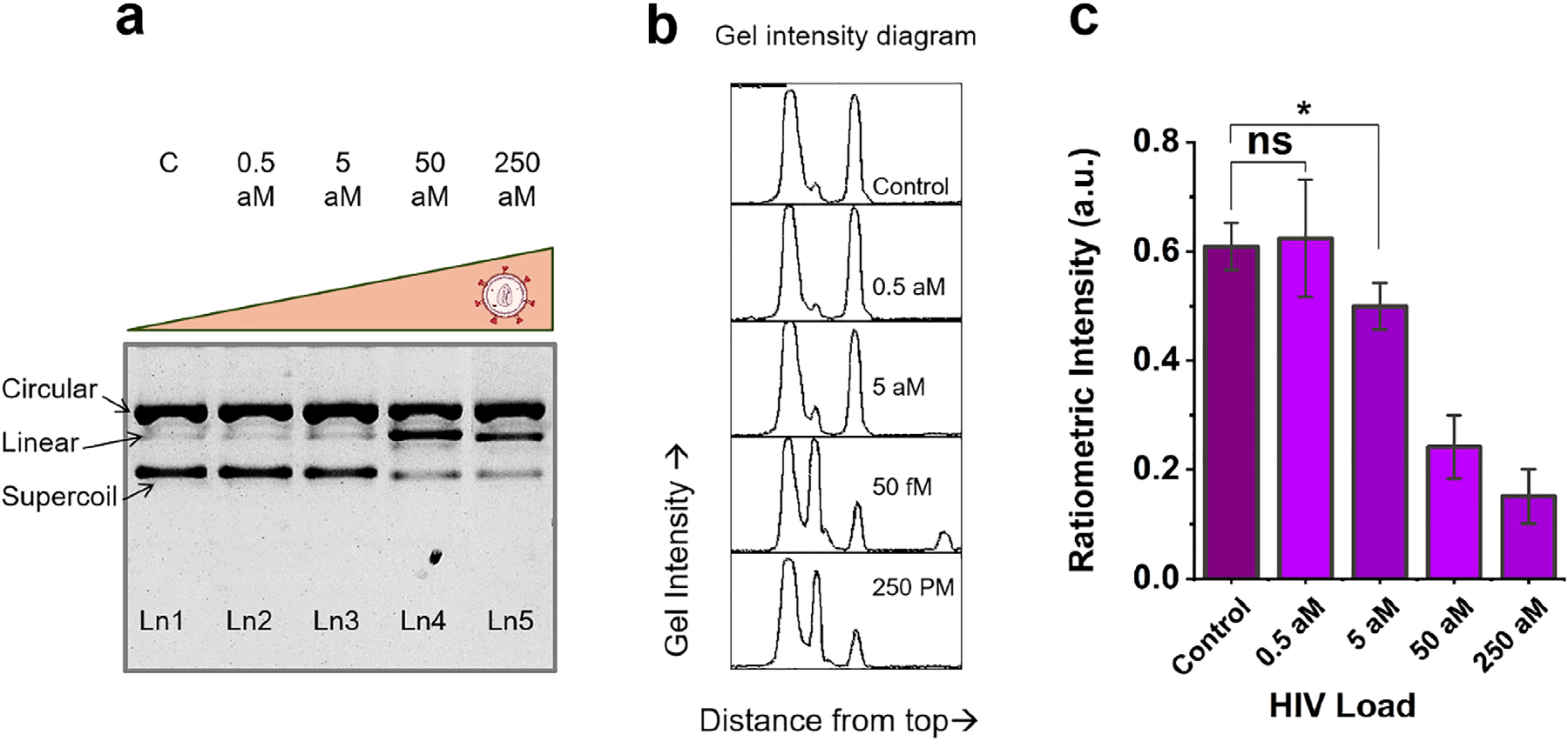
LOD quantification for the unfragmented HIV genome detection using RCA‐rCRISPR strategy. (a) Gel electrophoresis (1% agarose gel and 1×TBE buffer) results demonstrating RCA‐rCRISPR assay results for the unfragmented HIV genome detection. (b) Gel intensity diagrams of each lane in a. (c) LOD quantification for unfragmented HIV genome derived from PWH. Total reaction time was 3 h, of which 2 h were used for RCA and 1 h for rCRISPR. RCA was done at room temperature while rCRISPR was performed at 52°C. The graph shows statistical insignificance at *p* > 0.05 (ns), and significance at *p* < 0.05 (*). Abbreviations: c, negative control; aM, attomolar; Ln, lane; TBE, Tris‐borate EDTA; a.u., arbitrary unit.

After the initial demonstration of detecting an unprocessed HIV genome, we then quantified the limit of detection (LOD) of RCA‐rCRISPR for HIV derived from PWH. We serially diluted the samples in nuclease‐free water and ran the assay with 0.5 aM, 5 aM, 50 aM, 250 aM target RNA concentrations with a negative control (Figure 6). We ran gel electrophoresis to observe the supercoil relaxation of the plasmid reporters (Figure 6a). Then, we determined the LOD by measuring the ratiometric band intensity (Figure 6b). We observed that HIV viral load can be detected at a single‐digit aM level (5 aM, Figure 6c). It is worth mentioning that the LOD for unfragmented HIV genome was much better than the shorter synthetic targets (Figure 4e). It could be due to the more stable hybridization between the longer target and the padlock probe [57]. Besides, in shorter synthetic targets, end damage caused by light or oxygen exposure can reduce RCA extension efficiency [58]. In contrast, end damage doesn't affect the extension efficiency for longer targets since the long target first gets trimmed and RCA polymerization initiates immediately from cleaved ends. Moreover, longer targets likely exhibit higher binding affinity toward φ29, enhancing overall efficiency. The stronger interaction between an enzyme and long nucleic acid sequences is consistent with our previous observation that Cas12a binds more strongly to longer nucleic acids [42].

If we compared our results with a recently reported fluorescent reporting model that utilizes RCA‐rCRISPR‐Cas12a for RNA detection, we found that our low‐cost label‐free ratiometric reporting strategy performed two to three orders of magnitude better than the widely labeled F‐Q reporting system reported in the literature [30] (Table S2). Further, this ratiometric sensing strategy is advantageous from additional perspectives. Notably, DNA supercoil relaxation is rapid and can easily be evaluated using a more compact miniaturized gel electrophoresis system [59, 60] instead of a benchtop fluorescence microscope for POC detection, which is illustrated in the next section.

### 2.3 Development of a Smartphone‐Supported Mini Gel Electrophoresis System for POC RCA‐rCRISPR

Around 60% of PWH remain undiagnosed in resource‐limited, underserved communities. Therefore, it is crucial to develop an equipment‐free diagnostic system that can be deployed to such regions. The benchtop gel electrophoresis system requires an external power source, an expensive gel imager, and a computer to acquire gel images and analyze the data. Also, reagent consumption is high, which limits this strategy for POC use. Few previous studies demonstrated that microfluidic gel electrophoresis was feasible, but each system came with its own limitations. For instance, use of polymer‐based [61], glass‐based [62], and 3D‐printed [63] microfluidic gel electrophoresis has been reported, but these setups are for single‐lane gel and require high voltages (70–200 V). Although a low‐voltage microelectronic‐based gel electrophoresis [64] has been reported, the chip fabrication is very complicated.

We developed an all‐in‐one minigel electrophoresis system that requires a very low amount of power. This minigel system comprises a minigel cassette, a power supply (9 V batteries), one blue LED (Amazon, ∼3.21 lumens) to excite the gel during imaging, a fluorescence emission filter (1‐inch diameter, 550‐nm cutoff wavelength), and a smartphone holder for smartphone integration. To construct the minigel, a rectangular tank (2 cm × 2.5 cm × 0.75 cm) was printed using a 3D printer (Stratasys UPrint SE Plus). We installed two pieces of thin platinum wire (0.25 mm diameter) at the two opposite ends of the tank to make conductive electrodes. These electrodes were then connected to the battery using conductive wire. A blue LED was also connected to the same battery as the excitation light source. All construction steps, electrophoresis feasibility, preliminary demonstration, and system optimization steps have been shown in Figures S9–S12. This minigel device requires no external power, reduces the reagent consumption massively, and enables naked eye/smartphone detection.

**FIGURE 7:**
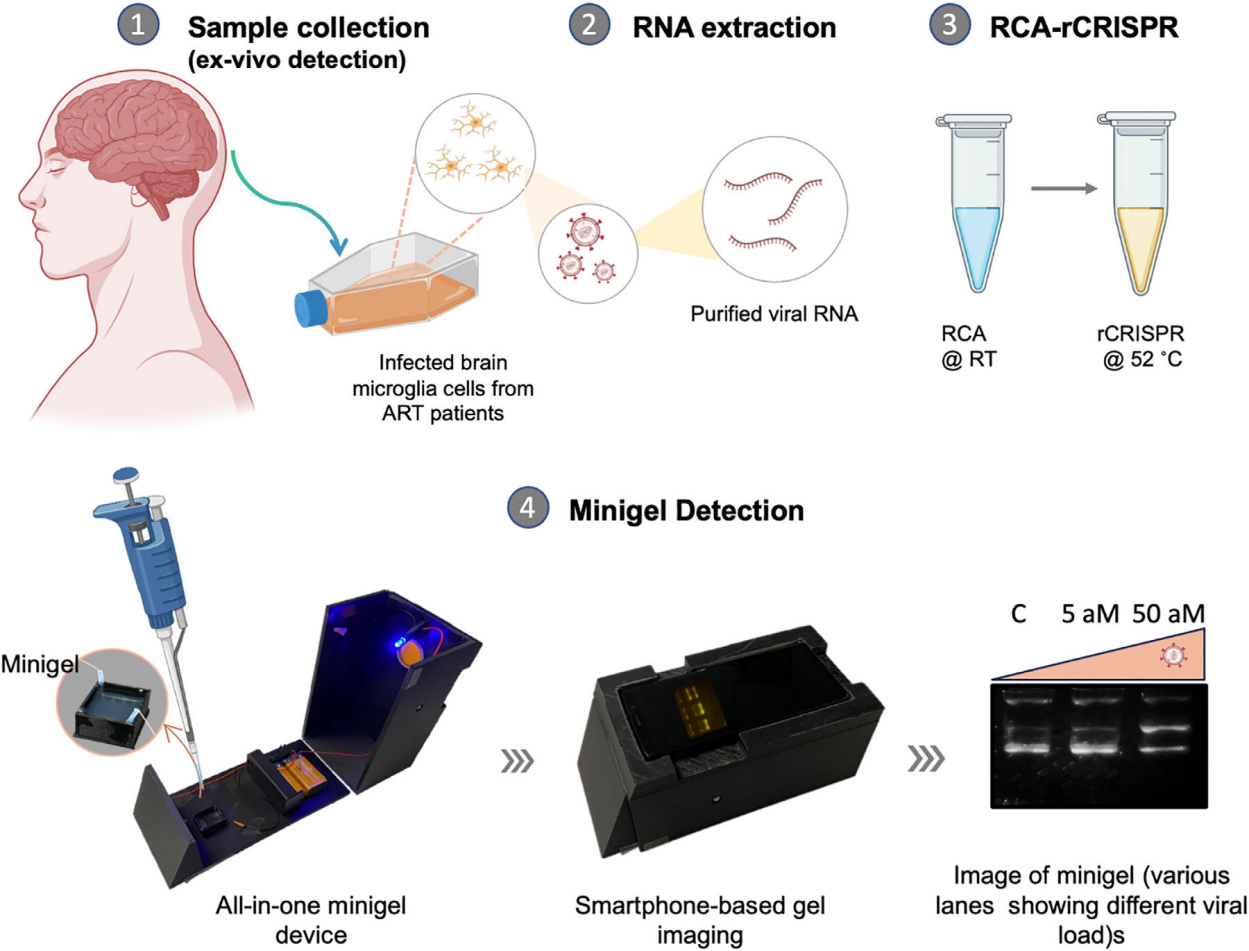
Detection of PWH‐derived HIV RNA at POC using the smartphone‐minigel system. Total reaction time was 3 h, of which 2 h were used for RCA and 1 h for rCRISPR. RCA was done at Room temperature while rCRISPR was performed at 52°C. Here, 5 aM and 50 aM correspond to viral loads of 3 and 30 copies/µL, respectively. Abbreviations: c, negative control; aM, attomolar.

We tested the performance of the smartphone‐minigel system to run the label‐free RCA‐rCRISPR for the detection of PWH‐derived HIV genomes (Figure 7). In the minigel device, 2 µL of RCA‐rCRISPR assay product was mixed with 1X loading dye and added into the holes in the minigel. After the completion of gel electrophoresis, a smartphone was placed on top of the device to capture the gel images under blue LED illumination. The 5 aM, 50 aM target concentrations, and a negative control were tested and compared. We observed that the supercoil band for 50 aM was significantly different (thinner) than the control band, meaning that our smartphone‐based minigel device was able to detect a very low amount of viral load with minimal instrumentation.

### 2.4 Point Mutation Detection With RCA‐rCRISPR

The RCA method is also known as a powerful technique that can be used to detect single nucleotide polymorphisms (SNPs) in DNA. As a model SNP target, we considered the *BRAF* gene (Figure S13) that encodes a protein called B‐Raf. In humans, mutations in the *BRAF* gene are associated with various cancers, particularly melanoma, colorectal cancer, and thyroid cancer. One of the most common mutations in the *BRAF* gene is the V600E mutation, where a valine (V) is substituted with glutamic acid (E) at position 600 of the protein. This mutation results in the constitutive activation of the *BRAF* protein kinase, leading to uncontrolled cell growth and proliferation, which is a hallmark of cancer [65]. In the domestic dog, the same base change, referred to as V595E, has a remarkably high frequency (85%) in canine urothelial carcinoma [46, 47]. We therefore chose to assess the use of our label‐free RCA‐rCRISPR strategy for sensitive detection of this single base change in the *BRAF* gene. In the presence of the wild‐type target, the 5′‐terminal with phosphate (p) and 3′‐terminal of the padlock probe join together by SplintR DNA ligase when the base at the 3′‐end of the padlock probe is perfectly matched to the DNA target. However, this circularization is hampered if there is a mismatch at the joining point. After that, the ligated padlock probe, the extended 3′‐end of the target gets trimmed with phi29 protein. Once the exonuclease activity is completed, the remaining sequence of the target acts as the primer for RCA amplification. The RCA amplification produced lots of amplicons, which further activated the CRISPR‐Cas12a system and resulted in the relaxation of supercoiled plasmid DNA reporters. This overall detection mechanism has been demonstrated in Figure S14a. The assay was performed with 25 fM of synthetic mutated DNA and wild‐type DNA (Figure S14b,c). In Figure S14c, we observed that while the supercoil band for the mutated DNA became significantly thinner (lane 3), the same band for wild type (lane 2) was comparable to the control band (lane 1). This result verifies the robustness of our assay platform for point mutation detection.

This study developed a new RNA detection method using a label‐free ratiometric CRISPR‐Cas12a technique using a recently discovered plasmid reporter. We successfully detected the unfragmented HIV genome using RCA, whereas previously reported studies either detected short miRNAs or longer RNAs only after mandatory pretreatment steps (e.g., enzymatic treatment, ultrasonication) to shorten the target and introduce an additional RCA primer (Table S2) [66, 67, 68]. Besides, we achieved a detection sensitivity at a single‐digit aM level, which is 100–1000 times greater than the previously reported fluorescent RCA‐CRISPR‐Cas12a technique found in the literature [30] (Table S2). A portable, battery‐powered, smartphone‐based minigel electrophoresis system was also developed to quantify ratiometric signals, enabling on‐site testing without sophisticated equipment. Compared to lateral flow CRISPR assays [18, 69] and microfluidic digital CRISPR assays [27, 37], the all‐in‐one gel electrophoresis device for CRISPR detection is much easier to scale up and implement at the POC. This innovative, low‐cost (as shown in Table S3, the cost per reaction is approximately $1, which is nearly half the cost of fluorescent reporting systems), and minimally instrumentalized approach offers a promising solution for combating the ongoing HIV pandemic in resource‐limited settings.

However, several challenges remain with the plasmid reporter and for the further development of the POC detection system. For instance, noticeable variations in gel patterns occurred. This had happened due to differences among the various batches of the ΦX174 plasmid, which could potentially be solved by sourcing or producing a near 100% supercoil plasmid. For instance, DNA gyrase could be utilized to increase the proportion of supercoiled DNA [44] (potentially close to 100%) in the initial sample, thereby establishing a high starting ratiometric value for improved contrast and a more consistent control band. For POC detection, sample preparation (extracting HIV viral RNA) still relies on lab‐centered techniques (e.g., commercial nucleic acid extraction methods). To simplify the nucleic acid extraction step, nuclease deactivation and heat treatment could be incorporated in future detection workflows [39, 70]. Besides, we only detected PWH‐derived samples ex vivo in this study. Future efforts should focus on directly detecting viral loads from patients’ plasma. Finally, detecting HIV in minimally equipped areas also poses regulatory challenges, because handling HIV samples typically requires a BSL‐2+/BSL‐3 laboratory [71], which would increase the cost and limit accessibility. In that respect, better and safer sample collection and storage protocols or methods will be needed to collaborate with the new diagnostic approaches.

## 3 Conclusions

In summary, this study demonstrated a POC friendly RCA‐rCRISPR system for sensitive HIV RNA detection, with four unique and useful aspects: First, it shows plasmid reporter can be combined with powerful CRISPR reaction schemes (e.g., RCA‐rCRISPR‐Cas12a) for label‐free RNA detection. Second, the molecular sizing‐based RCA‐rCRISPR assay was at least 100 times more sensitive than those previously demonstrated with fluorescent RCA‐CRISPR systems, which detected PWH‐derived HIV down to ∼3000 copies/mL (5 aM) ex vivo. Considering that the HIV viral load ranges from 15 000 to 50 000–200 000 copies/mL [72] during acute and chronic infection in PWH, our RCA‐rCRISPR detection of HIV is sensitive enough to capture active HIV infection in clinics. Third, unlike conventional RCA‐CRISPR, where ultrasonication or additional priming is needed for detecting larger targets, we demonstrated the detection of the long HIV genome without any nucleic acid fragmentation step, which simplified the assay. Fourth, a battery‐powered, smartphone‐based all‐in‐one minigel electrophoresis system was also developed to quantify RCA‐rCRISPR signals in an equipment‐free manner. Together, this straightforward yet sensitive strategy could be the gateway to accelerate the dissemination of POC HIV tests to resource‐limited communities, which is direly needed for early detection of HIV infection with a smartphone to prevent the pandemic globally.

## Author Contributions

Q.W. and N.M. initiated and conceived the project. N.M. performed all the experiments, collected, and analyzed the data. A.V. and S.Z. designed the cellphone holder. Y.T. and G.J. prepared HIV ex vivo patient samples. H.C. performed characterization of HIV samples using conventional assays such as ddPCR. A.S. and L.H. helped run the gel experiments to produce triplicate data. A.D.P. and M.B. provided SNP mutation sequences. S.J. shared synthetic HIV target sequences. M.B. helped repeat several experiments for the manuscript revision. N.M. and Q.W. wrote the manuscript. All the authors helped revise the manuscript.

## Conflicts of Interest

The authors declare no conflicts of interest.

## Supporting information




**Supporting File**: advs76124‐sup‐0001‐SuppMat.docx.

## Data Availability

The data that support the findings of this study are available in the supplementary material of this article.
