# Supplementary material for: Attomolar Detection of HIV‐1 With Label‐Free RCA‐rCRISPR on Smartphone
Source: Adv Sci (Weinh). 2026 Sep 2:e19508. Online ahead of print. doi: 10.1002/advs.202519508 (PMC13538080; doi:10.1002/advs.202519508)
Supplement: Supplementary file 1 [file ADVS-9999-e19508-s001.docx]

**Supporting Information**

**Attomolar Detection of HIV-1 with Label-Free RCA-rCRISPR on Smartphone**

Noor Mohammad,^1,2^ Anastasiia Steksova,^1^ Yuyang Tang,^3^ Leyao Huang,^1^ Alireza Velayati^1^, Shengwei Zhang,^1^ Aditi Dey Poonam,^1^ Sina Jamalzadegan,^1^ Matthew Breen,^4,5^ Hongjie Chen,^3^ Mahsa Bagi,^1^ Guochun Jiang,^3^ and Qingshan Wei^1^*

1. Department of Chemical and Biomolecular Engineering, North Carolina State University, Raleigh, NC 27695, USA
2. Department of Chemical Engineering, Bangladesh University of Engineering and Technology, Dhaka 1000, Bangladesh
3. UNC HIV Cure Center and Department of Biochemistry & Biophysics, University of North Carolina at Chapel Hill, NC 27599, USA
4. Department of Molecular Biomedical Sciences, College of Veterinary Medicine, North Carolina State University, Raleigh, NC 27607, USA
5. Comparative Medicine Institute, North Carolina State University, Raleigh, NC 27695, USA

* Corresponding email: qwei3@ncsu.edu

**Methods**

**Reagents and apparatus:** LbaCas12a, crRNA, synthetic RNA targets, and crRNA were purchased from Integrated DNA Technologies (IDT, Coralville, IA, USA). NEBuffer™ r2.1, and ΦX174 DNA (Catalog# FERSD0031) were purchased from Thermo-fisher. Ultrapure water (18.3 MΩ cm) was produced by the Milli-Q system (Millipore, Inc., USA) and used throughout the experiments. The sequences of ssDNA oligonucleotides and crRNA molecules are listed in Table S1.

**Label-free rCRISPR (ratiometric) assays for DNA detection:** All DNA and RNA oligos were stored in IDTE buffer (pH 8) at 10 μM concentrations in a -20 °C refrigerator and pre-warmed at 37 °C for 20 min before mixing. Reporter molecules (e.g., ΦX174) were diluted to 50 ng/µL in IDTE buffer (pH 8) and stored at 4°C for use throughout the experiments. For label-free rCRISPR assay, analyte solutions with different concentrations of ssDNA were added into the CRISPR reaction, consisting of 40 nM LbaCas12a, 20 nM gRNA, 4 ng/ µL reporter molecule, and 1X reaction buffer (e.g., 50 mM NaCl, 10 mM Tris-HCl, 10 mM MgCl_2_, 100 µg/ml recombinant albumin, pH=7.9 @ 25°C). The total reaction volume was 40 µL. The reaction was performed at 52°C for 1 hr. Gel electrophoresis was performed to observe the change in conformation of the plasmid reporter. 1% agarose gels were prepared using 1× TBE (Tris Borate EDTA) and 1× SYBR™ Safe DNA Gel Stain (Thermo-fisher, catalog# S33102). 10 μL of different reaction products with loading dye (5:1, v/v) were added to each well. Electrophoresis was done at 120 V for 30 min in the same buffer at room temperature. Finally, the agarose gels were scanned and recorded by the E-Gel Imager system (Invitrogen, USA).

**Label-free rCRISPR assays for RNA detection:** For RNA detection, three different mixtures were prepared, A: 100 nM padlock mixed with various concentrations of RNA target in 1x SplintR buffer; B: 400 units/mL SplintR ligase (catalog# M0375S), 125 units/mL phi29 polymerase (catalog# M0269S), 0.5 mM dNTPs, and 250 ng/uL recombinant albumin in 1.25X SplintR ligase buffer; C: 225 nM LbCas12a with 112.5 nM crRNA in 1X NEB r2.1 buffer. For one pot assay, A, B, and C were added in in 1:4:1 ratio. Then 4 ng/ µL label-free plasmid reporter was added. For two-pot assay, the same amount of reagents was added only difference is that mixture C was added after a 2-hr incubation of ‘A+B’. Total reaction time was 3 hours, of which 2 hours were used for RCA and 1 hour for rCRISPR. Then, gel electrophoresis was performed to evaluate the assay performance.

**Determination of HIV RNA (derived from PWH) by RT-digital droplet PCR (RT-ddPCR) and p24 by ELISA:** HIV RNA and p24 were measured as we previously reported^[1]^. Briefly, viral RNA was extracted from microglia culture supernatants using the QIAamp Viral RNA Mini Kit (Qiagen). The extracted RNA was treated with DNase I, and complementary DNA (cDNA) was synthesized using the SuperScript IV First-Strand Synthesis System (Life Technologies).

For the ddPCR assay, the reaction mixture (22 µL total volume) contained 11 µL of 2× ddPCR™ Supermix for Probes (Bio-Rad), 200 nM of each primer, 400 nM of probe mix, and 8 µL of cDNA. HIV-specific primers and probe targeting the gag region were used to quantify HIV RNA. Droplet generation and amplification were performed using the QX200 AutoDG Droplet Digital PCR System (Bio-Rad) in triplicate. Droplets were subsequently read and analyzed with QuantaSoft software in absolute quantification mode.

HIV p24 antigen levels in culture supernatants were measured using a p24 ELISA kit (ZeptoMetrix RETROtek HIV-1 p24 Antigen ELISA, Thermo Fisher Scientific) according to the manufacturer’s instructions.

**Detection of HIV genome derived from PWH:** As most HIV-positive individuals in the U.S. are under effective ART and typically have undetectable plasma viral loads, we performed ex vivo infection in primary brain microglia with HIV isolated from PWH (PMID: 37317962, donor 2), who was virally suppressed by ART. Two days post-infection, cultures were extensively washed to remove residual input virus. Supernatants from the infection culture were subsequently collected 7 days post-infection for HIV RNA detection in this study. The virus in the supernatant was inactivated and lysed using 1% Triton X-100, followed by viral RNA extraction using the Zymo Viral RNA Kit (Catalog #R1035), according to the manufacturer’s instructions. RNA was eluted in 15 µL of DNase/RNase-free water.

**Equipment-free *ex vivo* HIV RNA detection using smartphone readout minigel platform:** A piece of precast gel (1% agarose gel and 1× TBE buffer; gel size: ~1×1.5×.5 cm3) was placed in the minigel tank. 2 µL of reaction including 1× loading dye was added to each lane and run for 1 hr in the battery-powered minigel device. After that, a blue LED switch was turned on, and an image was captured using a smartphone (Samsung Galaxy S9).

**Electrophoresis analysis:** The gel results underwent analysis using ImageJ software. Initially, the background of the gel was removed. Subsequently, the intensity along the length of each lane was averaged across its width and graphed. Gel intensity was determined by identifying distinct peaks corresponding to supercoiled and relaxed circular conformations of DNA. Ratiometric intensities, representing the ratio of supercoiled DNA to relaxed circular DNA, were derived by dividing the intensity of the supercoiled band by that of the relaxed circular band. Finally, these ratiometric intensities were plotted against the target concentration.

**Statistical analysis:** The student's t-test was conducted using repeated experimental data (n=3) to compute p-values, thereby assessing the distinction between experimental outcomes and control groups and establishing the limit of detection (LOD). The graphical representation illustrates statistical insignificance when p>0.05 (ns) and statistical significance when p<0.05 (*).

**Nonfluorescent label-free rCRISPR for RNA detection**

**Fig. S1:** Relaxation of ΦX174 plasmid by CRISPR-Cas12a. (a) Cartoon illustration showing how activated Cas12a nicks the supercoil ΦX174 and relaxes it to convert it into circular form. (b) Gel electrophoresis (1% agarose gel and 1×TBE buffer) results demonstrating rCRISPR assay performed at 37 ˚C for 1 hr. Ln1: negative control; Ln2-3: increasing concentrations of target. Abbreviations: Ln, lane; TBE, Tris-borate EDTA; kb, kilo base-pair; pM, picomolar.

**Fig. S2:** Intensity plot of rCRISPR assay (gel image shown in Fig. 2d). Abbreviations: pM, picomolar; nM, nanomolar.

**Fig. S3:** RCA-rCRISPR assay for detecting RNA target. (a) Gel electrophoresis (1% agarose gel and 1×TBE buffer) results demonstrating 2-hr RCA initiated CRISPR-Cas12a induced trans-nicking of phix174 for various target concentrations. (b) Schematic showing how SplintR could revert DNA supercoil relaxation. Abbreviations: c, negative control; pM, picomolar; nM, nanomolar; Ln, lane; TBE, Tris-borate EDTA.

**Fig. S4:** Effect of Padlock concentration. Gel electrophoresis (1% agarose gel and 1×TBE buffer) results demonstrating RNA detection using rCRISPR assay with (a) 25 nM (d) 50 nM, and (g) 250 nM padlock. (b, e, h) Intensity plot diagrams for each lane of the gel in a, d, and g. Bar chart showing assay results with (c) 25 nM (f) 50 nM, and (i) 250 nM padlock. Abbreviations: PL, padlock; c, negative control; pM, picomolar; fM, femtomolar; Ln, lane; TBE, Tris-borate EDTA.

**Fig. S5:** Possible mechanism of how higher padlock concentration inhibit RCA reaction. Abbreviations: PL, padlock; RCA, rolling circle amplification.

**Fig. S6:** Assay integration. Schematic showing experimental procedure (a) with ligase deactivation (c) without ligase deactivation, and (e) of one pot assay. (b, d, f) Intensity plot diagrams for each lane of the gel in Fig. 4a, 4d, and 4g. Abbreviations: pM, picomolar; RCA, rolling circle amplification.

***Ex Vivo* HIV detection using the rCRISPR technique**

**Fig. S7:** Quantification of HIV load (derived from PWH) with conventional assay methods. (a) Quantification of HIV viral genome was done by RT-digital droplet PCR (ddPCR). (b) p24 antigen quantification by ELISA.

**Fig. S8:** Evaluation of false positives. (a) Off-target checking tool: Cas-OFFinder. (b) Nicking endonuclease and DNase contamination evaluation. Abbreviations: RCA, rolling circle amplification.

**Towards POC detection (equipment-free detection)**

**Fig. S9:** Fabrication of minigel cassette. (a) AutoCAD drawing of minigel cassete. (b) Installation of electrodes using Pt wire. (c) Electrodes connection with a 9v battery.

**Fig. S10:** Operational testing of mini-gel using 9v battery. (a,c,e) Longer/narrow gel. (b,d,f) Shorter/wider gel. Abbreviations: Pt, platinum; V, voltage, I, current; kb, kilobase pair.

**Fig. S11:** System modifications. (a) Thinner gel resulted in no DNA band. (b) H2/O2 evolution pushed the gel to float. (c) New design that allows thicker gel. (d) phix174 DNA band detection after ratiometric CRISPR assay. Abbreviations: C, control; pM, picomolar.

**Fig. S12:** Gel visualization (without transilluminator). (a) Naked eye minigel DNA band detection using a blue light torch. (b) phix174 DNA band detection after ratiometric CRISPR assay. Abbreviations: C, control; pM, picomolar.

**Universal detection (DNA mutation)**

**Fig. S13:** SNP location of the canine *BRAF* mutation. (a) Mutated gene. (b) Wild type gene.

**Fig. S14**: SNP detection using RCA-rCRISPR strategy. (a) Assay mechanism showing how SNP can be detected with the RCA-initiated rCRISPR technique. (b) DNA sequences (red colored ‘T’ is the point mutation) used for detection. (c) Gel electrophoresis (1% agarose gel and 1×TBE buffer) results demonstrating the rCRISPR assay result for SNP detection. Abbreviations: RCA, rolling circle amplification; RNP, ribonucleoprotein; WT, wild type; MUT, mutation; Ln, lane; fM, femtomolar; TBE, Tris-borate EDTA.

**Table S1. Oligos used for the study.**

| Item | Sequence (5’-3') |
| --- | --- |
| gRNA | UAAUUUCUACUAAGUGUAGAUCGUCGCCGUCCAGCUCGACC |
| Amplicon (ssDNA) | GGT CGA GCT GGA CGG CGA CG |
| Padlock_RNA | /5Phos/CTG ATA AGC TAA GAT ACC CTA ACC ATC GAT TTA CGT CGC CGT CCA GCT CGA CCT CAA CAT CAG T |
| Padlock_HIV | /5Phos/CCC TGT GGA AGA CCC TAA CCA TCG ATT TAC GTC GCC GTC CAG CTC GAC CAT CCT TTC CAT |
| Padlock_dogMut | /5Phos/AGA AAT CTC GAA CCC TAA CCA TCG ATT TAC GTC GCC GTC CAG CTC GAC CTC TAG CCA CAG |
| RNA | UAGCUUAUCAGACUGAUGUUGA |
| HIV synthetic target | GUGCUUCCACAGGGAUGGAAAGGAUCAC |
| DogMut | ACT GTT CAA ACT GAT GGG ACC CAC TCC ATC GAG ATT TCT CTG TGG CTA GAC CAA AAT CAC CTA TTT TTA CTG TGA GGT CTT CAT G |
| DogWT | ACT GTT CAA ACT GAT GGG ACC CAC TCC ATC GAG ATT TCA CTG TGG CTA GAC CAA AAT CAC CTA TTT TTA CTG TGA GGT CTT CAT G |

**Table S2. Comparative Summary of RCA-Based Techniques for RNA and DNA Detection**

| Assay Type | Target | Pretreatment Process | | LOD | Reporter type | Assay time | POC | Ref |
| --- | --- | --- | --- | --- | --- | --- | --- | --- |
| Cas12a-SCR  (RCA with CRISPR-Cas12a) | Short miRNA (miR-21) | - | 47 fM | | Labeled fluorescent reporter | 6h | no | ^[2]^ |
| Cas12a-TCA  (H-RCA with CRISPR-Cas12a) | Short miRNA (miR-21) | - | Single digit fM | | Labeled fluorescent reporter | 7h | no | ^[3]^ |
| CRISPR-Cas12a-enhanced RCA | Short miRNA (miR-21) | - | 35 fM | | Labeled fluorescent reporter | 4.5 h | no | ^[4]^ |
| RCA-assisted CRISPR/Cas9 cleavage | Short miRNA (miR-21) | - | 90 fM | | Fluorogenic readout by Cas9 cleavage | 3.5 h | no | ^[5]^ |
| RCA-fluorescent CRISPR | Short miRNA | - | Single digit fM level | | Labeled fluorescent reporter | 1.5 hr | yes | ^[12]^ |
| RCA-fluorescent CRISPR | circRNA | Additional primers | 300 aM | | Fluorescent | <2 hr | no | ^[11]^ |
| RCA-CRISPR- SERS Nanotag | Exosomal miRNA-21 | - | 0.62 aM | | SERS | - | no | ^[10]^ |
| RCA | Long RNA sequence | RNase H-assisted RNA-primed RCA | pM range | | Fluorescent | 2 hr | no | ^[6]^ |
| RCA | Whole genome, viral RNA | DNAzyme-Mediated RNA Cleavage | 0.5 fM | | Fluorescent | 2.5 hr | no | ^[7]^ |
| RCA | Longer DNA (mutation detection) | Additional primers | 70 fM (wild type to the mutant is 10,000:1 ) | | Colorimetric | 2.5 hr | no | ^[8]^ |
| RCA | Longer DNA (mutation detection) | Additional primers | Wild type to the mutant is 10:1) | | - | ~2 hr | no | ^[9]^ |
| RCA-rCRISPR | **Whole RNA genome** (also short RNA) | **No treatment required** | **Single digit aM level** | | **Label-free reporting** | ~2.5 hr | **yes** | This work |

**Table S3. Comparative Cost Estimation of Fluorescent and rCRISPR methods**

| **Items** | | **RCA-fCRISPR** *(F-Q reporter)* | **RCA-rCRISPR**  *(ΦX174 reporter)* |
| --- | --- | --- | --- |
| Reporter cost | Cost per order ($, IDT) | 326 | 320 |
|  | Amount per order (After purification) | 6.5 (nmole) | 150 (ng) |
|  | Consumption per reaction | 0.024 (nmole) | 20 (ng) |
|  | Total number of reactions per order | 271 | 7,500 |
| **Reporter cost per 100 reactions ($)** | | **120.37** | **4.27** |
| **Other RCA-CRISPR reagent (Cas12a, SplintR, polymerase, dNTP, etc.) cost per 100 reactions ($)**^[12]^  *(excluding reporter cost)* | | **80** | **80** |
| **Signal quantification cost (approximate) per 100 reactions ($)** | | **5** | **15** |
| **Total cost per 100 reactions ($)** | | **205.37** | **99.27** |

References:

[1]	Y. Tang, A. Chaillon, S. Gianella, L. M. Wong, D. Li, T. L. Simermeyer, M. Porrachia, C. Ignacio, B. Woodworth, D. Zhong, *The Journal of clinical investigation* **2023**, *133*.

[2]	G. Wang, W. Tian, X. Liu, W. Ren, C. Liu, *Analytical Chemistry* **2020**, *92*, 6702-6708.

[3]	W. Tian, X. Liu, G. Wang, C. Liu, *Chemical Communications* **2020**, *56*, 13445-13448.

[4]	G. Zhang, L. Zhang, J. Tong, X. Zhao, J. Ren, *Microchemical Journal* **2020**, *158*, 105239.

[5]	R. Wang, X. Zhao, X. Chen, X. Qiu, G. Qing, H. Zhang, L. Zhang, X. Hu, Z. He, D. Zhong, *Analytical chemistry* **2019**, *92*, 2176-2185.

[6]	H. Takahashi, M. Ohkawachi, K. Horio, T. Kobori, T. Aki, Y. Matsumura, Y. Nakashimada, Y. Okamura, *Scientific reports* **2018**, *8*, 7770.

[7]	J. Gu, A. Mathai, C. Nurmi, D. White, G. Panesar, D. Yamamura, C. Balion, J. Gubbay, K. Mossman, A. Capretta, *Chemistry–A European Journal* **2023**, *29*, e202300075.

[8]	J. Li, T. Deng, X. Chu, R. Yang, J. Jiang, G. Shen, R. Yu, *Analytical chemistry* **2010**, *82*, 2811-2816.

[9]	P. M. Lizardi, X. Huang, Z. Zhu, P. Bray-Ward, D. C. Thomas, D. C. Ward, *Nature genetics* **1998**, *19*, 225-232.

[10]	J. Teng, Y. Chen, W. Zhang, H. Xu, L. Ke, H. Xu, J. Wang, *Analytical chemistry* **2025**, *97*, 21098-21105.

[11]	X. Ke, A. Liang, C. Chen, T. Hu, *Analytical Methods* **2024**, *16*, 3256-3262.

[12]	H. Yan, Y. Wen, Z. Tian, N. Hart, S. Han, S. J. Hughes, Y. Zeng, *Nat Biomed Eng* **2023**, *7*, 1583-1601.
